# Tranexamic Acid Versus Epinephrine as Submucosal Injectate During Endoscopic Mucosal Resection of Large Colorectal Lesions: A Randomized Controlled Trial

**DOI:** 10.3390/jcm15134969

**Published:** 2026-06-25

**Authors:** Anton Bermont, Daniel L. Cohen, Daniela Malkin, Ariel Ben Shimol, Shay Matalon, Haim Shirin, Sergei Vosko

**Affiliations:** 1 Gonczarowski Family Institute of Gastroenterology, Hepatology and Nutrition, Shamir Medical Center (Assaf Harofeh), Zerifin 703301, Israel; docdannycohen@yahoo.com (D.L.C.); danielalevy7@hotmail.com (D.M.); arielbenshimol@gmail.com (A.B.S.); shaimatalon@yahoo.com (S.M.); haimsh@shamir.gov.il (H.S.); 2 Gray Faculty of Medical and Health Sciences, Tel Aviv University, Klatzkin St 35, Tel Aviv-Yafo 6997801, Israel; 3 Department of Gastroenterology, Hadassah Medical Center, Jerusalem 12000, Israel; sergeivosko@gmail.com

**Keywords:** tranexamic acid, endoscopic mucosal resection, colorectal polyps, delayed post-polypectomy bleeding, submucosal injection, epinephrine, post-procedural pain, hemostasis, randomized controlled trial, advanced endoscopy

## Abstract

**Background and Aims:** Bleeding and post-procedural pain are common adverse events after endoscopic mucosal resection (EMR) of large colorectal lesions. Epinephrine is frequently added to submucosal injectates, although its benefit for delayed bleeding is inconsistent and associated with post-procedural pain. Tranexamic acid (TXA), an antifibrinolytic agent used to prevent bleeding in several medical conditions, has not been evaluated during colorectal EMR. We compared TXA with epinephrine for bleeding outcomes, safety, and post-procedural pain. **Methods:** In this prospective, double-blind, randomized controlled pilot trial, adults undergoing EMR for colorectal lesions ≥20 mm were randomized 1:1 to receive a TXA-containing or epinephrine-containing submucosal injectate. Primary outcomes were intraprocedural bleeding severity and delayed bleeding. Secondary outcomes included post-procedural pain, post-polypectomy syndrome, and thromboembolic events. **Results:** The study included 121 patients (TXA, n = 60; epinephrine, n = 61). Intraprocedural bleeding rates and severity were similar between groups, and hemostasis was achieved endoscopically in all cases. Delayed bleeding occurred in 8.3% of patients in the TXA group and 11.5% in the epinephrine group (*p* = 0.762); clinically significant delayed bleeding occurred in 6.7% and 6.6%, respectively. Lesion size was the only independent predictor of delayed bleeding. Post-procedural pain was significantly less frequent with TXA (5.0% vs. 39.3%; *p* < 0.001), and no TXA-treated patients required opioid analgesia compared with 18% in the epinephrine group (*p* < 0.001). No major adverse events were observed in the TXA group. **Conclusions:** In this pilot study, TXA achieved bleeding outcomes comparable to epinephrine while significantly reducing post-procedural pain in large colorectal EMR procedures, warranting further evaluation in larger trials.

## 1. Introduction

Endoscopic mucosal resection (EMR) is an established technique for the removal of large non-pedunculated colorectal lesions and enables organ-preserving treatment of superficial neoplasia [1]. Despite ongoing technical refinements, bleeding remains the most frequent adverse event, with intraprocedural and delayed bleeding (DB) rates reported to be as high as 11% [1,2]. Such events may require endoscopic hemostasis, hospitalization, transfusion, or repeat intervention, thereby increasing procedural complexity and patient morbidity. A range of hemostatic strategies—including soft coagulation using the snare tip [3,4], epinephrine injection, and hemoclip placement [5]—is routinely applied to mitigate bleeding risk or manage active bleeding.

Epinephrine is often incorporated into submucosal injection solutions because of its vasoconstrictive effect [1]. However, systemic absorption may lead to hypertension, tachyarrhythmias, and increased post-procedural pain [6]. Notably, a recent randomized trial suggested that epinephrine-containing injectates may significantly contribute to post-EMR pain [7]. In Israel, diluted epinephrine has traditionally been included in submucosal injection solutions for EMR and remains the routine standard for immediate intraprocedural hemostasis, despite limited evidence supporting its effect on DB [8,9].

Interestingly, preliminary experimental data from a small in vivo porcine endoscopic submucosal dissection (ESD) model have suggested that the use of hemostatic adjuncts, including epinephrine and tranexamic acid, may be associated with reduced tissue injury at the resection site [10].

Tranexamic acid (TXA), a synthetic antifibrinolytic agent, has a longstanding safety record and is widely used across surgical disciplines. By inhibiting fibrinolysis and stabilizing fibrin clots, TXA effectively reduces intraoperative blood loss and transfusion requirements without increasing thromboembolic risk in most clinical settings [11,12,13,14,15,16]. Topical TXA may offer further benefit, achieving high local concentrations with minimal systemic exposure. Its topical application has demonstrated efficacy in dermatologic, orthopedic, and gynecologic surgery, with emerging evidence suggesting a reduction in postoperative pain as well [17,18,19,20,21,22].

The theoretical advantages of TXA over epinephrine in the context of EMR include improved hemostasis, avoidance of vasoconstriction-associated systemic adverse events, and reductions in post-procedural pain. However, TXA has not been systematically evaluated as an adjunct to submucosal injectate during colonic EMR. We, therefore, conducted this exploratory pilot trial hypothesizing that TXA would achieve bleeding outcomes comparable to epinephrine, while potentially reducing other adverse events. The objective of this prospective, double-blind, randomized controlled pilot study was to compare TXA versus epinephrine as adjuncts to submucosal injection during large colonic EMR.

## 2. Materials and Methods

**Study design and participants**: This prospective, double-blind, randomized pilot and feasibility study was conducted at a single tertiary academic medical center. Patients were randomly assigned in a 1:1 ratio to receive either a TXA containing injectate or an epinephrine containing injectate during EMR. Both endoscopists and patients were blinded to group allocation. Trial was approved by the Institutional Review Board (ASF-0017-22). All participants provided written informed consent before enrollment. The trial was registered prior to the start of patient recruitment in May 2022 at ClinicalTrials.gov (Identifier: NCT05345613).

Eligible participants were adults aged 18 years or older with colonic lesions ≥20 mm scheduled for outpatient EMR, including non-neoplastic or neoplastic superficial lesions deemed suitable for endoscopic removal. Patients with a known allergy to TXA, a history of seizures, or pregnancy were excluded. Anticoagulants and antiplatelet medication were required to be withheld prior to the EMR procedure according to guidelines, except for aspirin which was allowed to be continued.

**Randomization, procedure and post procedure follow up**: A research assistant not involved in patient care prepared identical syringes for all procedures, thereby ensuring allocation concealment and preventing clinicians and participants from identifying group assignment. Participants were enrolled by the treating physicians, while group allocation was performed by an independent research assistant who had no role in patient management or outcome assessment.

The TXA injectate consisted of 9 mL of a standard solution (indigo carmine mixed with 4% succinylated gelatin) combined with 1 mL TXA (100 mg), whereas the control injectate consisted of the same standard solution mixed with 1 mL epinephrine (1:100,000). Submucosal injection was performed according to the endoscopist’s discretion to achieve an adequate mucosal lift.

Standard EMR technique was used in all cases. Throughout the procedure, lesion characteristics, intraprocedural events, injection volumes, and the severity of bleeding were recorded. Bleeding was categorized as “mild” when self-limited or easily controlled without interrupting the procedure, “moderate” when persistent and requiring endoscopic hemostasis with transient impairment of visualization, and “severe” when profuse enough to markedly obscure the visual field and necessitate coagulation forceps use, temporary cessation of the procedure for more than 2 min, or prolonged hemostatic intervention. Deep mural injury and perforation were likewise documented. All EMR procedures were performed by a senior gastroenterologist with expertise in advanced endoscopy.

Prior to discharge, patients were evaluated in the recovery room. Post-procedure pain was assessed using a numerical rating scale (NRS) ranging from 1 to 10. Pain was assessed 45–60 min after the procedure in the recovery room by a blinded recovery-room nurse. Scores < 7 were classified as mild to moderate pain, whereas scores ≥ 7 were classified as severe pain. Opioid analgesia (fentanyl) was administered for patients with severe pain. The need for opioid analgesia was recorded separately as a distinct outcome.

Patients were subsequently evaluated in the outpatient clinic setting to assess for DB adverse events, including bleeding, abdominal pain, or the need for hospitalization. Histopathologic results and findings from surveillance colonoscopy were subsequently obtained from the medical record.

**Study outcomes**: The primary outcome was the rate of intraprocedural bleeding, classified as none, mild, moderate, or severe, and rate of DB. Secondary outcomes included post-procedural pain, the need for opioid analgesia, deep mural injury or perforation, post-polypectomy syndrome, hospitalization, and thromboembolic events, as well as lesion recurrence detected at 6-month surveillance colonoscopy.

**Statistical analysis**: As this was a pilot study, no formal sample size calculation was performed; the sample size was based on feasibility and reflected the number of eligible patients available for enrollment during the study period. Quantitative variables were analyzed using Student’s *t*-test or the Mann–Whitney U test, depending on distribution, and categorical variables using Pearson’s χ^2^ test. Multivariable logistic regression was performed to identify independent predictors of DB. No important changes to the trial protocol, outcomes, or statistical analyses were made after trial commencement. All outcomes and analyses were prespecified in the study protocol. Analyses were performed on a modified intention-to-treat basis. Missing data were minimal and were not imputed. A two-sided *p*-value < 0.05 was considered statistically significant. Ninety-five percent confidence intervals for differences in proportions were calculated using the Wald method. All analyses were conducted using SPSS software, version 26.0 (IBM Corporation, Armonk, NY, USA).

## 3. Results

### 3.1. Study Population

**Figure 1:**
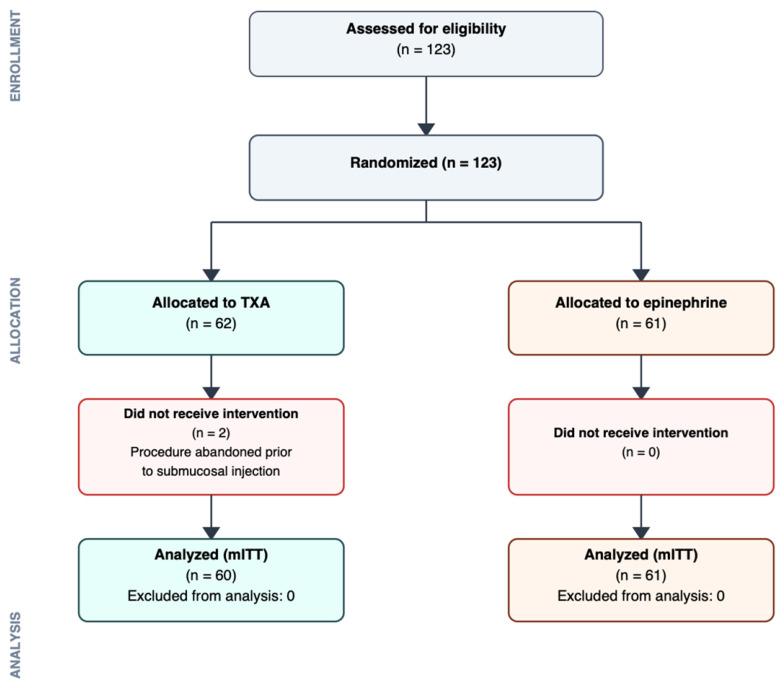
Trial flow diagram. mITT = Modified intention-to-treat; TXA = Tranexamic acid.

A total of 123 patients were enrolled. Two patients were subsequently excluded as their lesions were not amenable to EMR. In one case, the lesion showed endoscopic features suggestive of adenocarcinoma, and resection was therefore not attempted; the patient was referred for surgery. In the second case, the polyp demonstrated deep invasion into the appendix, precluding complete endoscopic removal, and the patient was likewise referred for surgical management. These two participants were excluded from the final analysis, resulting in 121 patients (60 TXA, 61 epinephrine) included in the modified intention-to-treat analysis (Figure 1). A modified intention-to-treat approach was used because these two patients did not undergo the allocated EMR intervention and were therefore not evaluable for the procedure-related outcomes.

Despite randomization, some baseline characteristics differed between the groups (Table 1). The mean age was 69 years, with patients in the TXA arm slightly older than those in the epinephrine arm (median 71 vs. 67 years; *p* = 0.014). Sex distribution, smoking status, and most comorbidities were comparable. Body mass index (BMI) was modestly higher in the TXA group (28 vs. 26.4; *p* = 0.045), and ischemic heart disease was more frequent among TXA-treated patients (20% vs. 6.6%; *p* = 0.034).

Antithrombotic use was similar between groups (Table 2). Anticoagulants were used in 10.7% of patients overall and were withheld before EMR according to guideline recommendations. Continuous antiplatelet therapy was more common in the TXA group, with 19 patients (31.7%) continuing aspirin during the procedure compared with eight (13.1%) in the epinephrine group (*p* = 0.014).

### 3.2. Colorectal Lesion Characteristics

Lesion characteristics are shown in Table 3. Median lesion size was 30 mm (IQR 25–40) in both groups. No significant differences were observed in lesion location, Paris classification, morphology, Japan NBI Expert Team (JNET) classification, or histopathologic diagnosis. The rate of malignant polyps was identical (5% in each group).

Data on prior biopsy, previous resection attempts, fibrosis, and technical parameters are summarized in Table 4. Severe fibrosis was more common in the TXA group (16.7% vs. 4.9%; *p* = 0.044), while other variables were similar. The median injection volume was 20 mL in both groups.

Among the six resected polyps classified as malignant on pathological examination, one patient underwent endoscopic surveillance only as the depth of invasion was limited. Another patient was lost to follow-up, although an interim computed tomography (CT) scan was normal. The remaining four patients were referred for surgery. Residual disease was found in one case, with a single positive lymph node and a 1 mm focus of carcinoma in the resection scar. No residual carcinoma or nodal involvement was detected in the other three patients.

### 3.3. Bleeding Outcomes

The primary outcome, intraprocedural bleeding, occurred at similar frequencies in both groups (*p* = 0.264). Severe bleeding was rare, occurring in one patient in the TXA arm and in none in the epinephrine group. Hemostasis was achieved in all cases, most often with snare-tip soft coagulation.

Delayed bleeding occurred in 8.3% of patients in the TXA group and 11.5% in the epinephrine group (*p* = 0.762; risk difference −3.1%, 95% CI −13.8% to 7.5%). Clinically significant delayed bleeding (CSDB)—defined as bleeding requiring emergency department evaluation or hospitalization—occurred in four patients in each group (6.7% vs. 6.6%; *p* > 0.999; risk difference 0.1%, 95% CI −8.7% to 9.0%). Outcome results are summarized in Table 5.

Multivariable logistic regression was performed to identify independent predictors of DB. Candidate predictors of DB (lesion size, location, age, BMI, continued antiplatelet therapy, injectate type, and severe fibrosis) were first assessed in univariable logistic regression. To avoid model overfitting given the limited number of DB events (n = 12), only two variables were entered into the multivariable model: lesion size (significant on univariable analysis) and lesion location (a recognized risk factor for post-EMR DB). In this model, only lesion size remained an independent predictor (OR 1.09 per mm, 95% CI 1.04–1.15; *p* = 0.001), while right-sided location showed a non-significant trend (OR 4.34, 95% CI 0.77–24.34; *p* = 0.095). The remaining variables were not associated with DB on univariable analysis and were not included in the multivariable model.

### 3.4. Post-Procedural Pain and Other Secondary Outcomes

Post-procedural pain was significantly less common in the TXA group (5.0% vs. 39.3%; *p* < 0.001), and none of these patients required opioid analgesia, compared with 18% in the epinephrine arm (*p* < 0.001). Deep mural injury or perforation occurred only in the epinephrine arm (6.6% vs. 0%; *p* = 0.119). Hospitalization or post-polypectomy syndrome occurred exclusively in the epinephrine group (6.6% vs. 0%; *p* = 0.119). Among patients who reported pain, the median NRS score was lower in the TXA group (4.0; IQR 3.5–5.0) than in the epinephrine group (6.5; IQR 4.8–8.0), and the overall distribution of pain scores differed significantly between groups (Mann–Whitney U test, *p* < 0.001).

No major adverse events related to tranexamic acid were observed. Specifically, no thromboembolic events, anaphylaxis, seizures, or other unexpected complications occurred either immediately after the procedure or throughout the follow-up period.

### 3.5. Polyp Recurrence

At the time of data analysis, 83 patients (68.6%) had completed 6-month surveillance colonoscopy. The remaining 38 patients (31.4%) either had not yet reached the 6-month follow-up timepoint or were lost to follow-up. Among those who completed surveillance, recurrence rates were numerically higher in the TXA group (6.5% vs. 0%; *p* = 0.250). Recurrence was detected in three patients, all of whom had undergone EMR for large lesions (35 mm, 70 mm, and 40 mm). Each case was associated with severe fibrosis, and two had a history of prior resection attempts. Two recurrences were managed successfully with snare resection and snare-tip soft coagulation. The third, in which biopsy revealed tubular adenoma with low-grade dysplasia arising from a fibrotic defect after a difficult EMR, was treated with full-thickness resection device. All three patients underwent follow-up colonoscopy, and no further recurrence was observed.

## 4. Discussion

Bleeding—both intraprocedural and delayed—remains the most important adverse event following EMR of large colorectal lesions. DB occurs in approximately 6–10% of cases, while CSDB affects 3–7% and has become the preferred endpoint because it more accurately reflects patient safety and healthcare utilization [2,23,24]. Numerous preventive techniques have been explored, including prophylactic clipping, thermal ablation of the resection base, self-assembling peptide matrices, and modified injection solutions; however, their effectiveness has been inconsistent across studies [4,25,26,27]. Among these approaches, prophylactic clipping currently provides the most reproducible benefit, with a randomized control trial demonstrating a reduction in post-EMR bleeding from 7.1% to 3.5% for large right-sided lesions [28]. Epinephrine, which is routinely used in submucosal injection solutions at many centers, has shown variable efficacy in preventing immediate bleeding and inconsistent effects on DB [8]. In addition, epinephrine has recently been associated with an increased incidence of post-polypectomy pain syndrome [7]. Together, these observations highlight the need for alternative strategies that may improve both bleeding outcomes and overall procedural tolerability.

One of the main findings of this randomized controlled trial was a substantial reduction in post-procedural pain and fewer ischemia-related complications in patients receiving TXA compared with epinephrine. Post-procedural pain occurred in 5.0% of patients in the TXA group versus 39.3% in the epinephrine group (*p* < 0.001), corresponding to an 87% relative risk reduction. This difference was statistically significant and clinically relevant. Furthermore, no patients in the TXA group required opioid analgesia, compared with 18% in the epinephrine arm (*p* < 0.001). In this cohort, all observed cases of deep mural injury, perforation, and post-polypectomy syndrome requiring hospitalization occurred in the epinephrine group, although event numbers were small and differences did not reach statistical significance.

The observed reduction in post-procedural pain with TXA compared with epinephrine may be related to differences in their pharmacologic profiles. Epinephrine induces local vasoconstriction, which may impair mucosal and submucosal perfusion during and after EMR, potentially contributing to tissue ischemia, inflammation, delayed healing, and pain syndromes, including post-polypectomy syndrome. In contrast, TXA does not possess vasoactive properties and exerts its hemostatic effect through inhibition of fibrinolysis, thereby allowing preservation of tissue perfusion during the healing process. Although mechanistic conclusions cannot be definitively drawn from this study, the absence of vasoconstriction-associated ischemia may partially explain the reduced pain observed in the TXA group. Thus, because the comparator injectate contained epinephrine, the lower pain frequency in the TXA arm may reflect avoidance of epinephrine-induced ischemic pain rather than a specific analgesic effect of TXA itself. A future study comparing TXA versus a saline (placebo) injectate would help clarify this.

With respect to bleeding outcomes, TXA was associated with bleeding rates comparable to those observed with epinephrine, despite the TXA group being older, sicker, and having more aspirin use. Intraprocedural bleeding occurred at similar frequencies in both groups and was successfully managed endoscopically in all cases. Most bleeding events were mild or moderate and self-limited, consistent with prior large EMR cohorts [24].

Although moderate intraprocedural bleeding was numerically more frequent in the TXA group (13.3% vs. 4.9%), this difference did not reach statistical significance.

Delayed bleeding rates were also similar between groups (8.3% vs. 11.5%, *p* = 0.762), as was clinically significant DB (6.7% vs. 6.6%, *p* > 0.999). These rates are consistent with those reported in previous large EMR studies of non-pedunculated colorectal lesions ≥20 mm. In the multicenter randomized trial by Pohl et al., severe post-EMR bleeding occurred in 7.1% of controls versus 3.5% with clip closure, with proximal colon lesions showing rates of 9.6% and 3.3%, respectively [29]. In Gupta et al., clinically significant post-EMR bleeding in right-sided lesions was 10.6% without clipping and 3.4% with prophylactic clipping [23]. Similarly, Bahin et al. reported CSDB rates of 8.0% in controls and 5.2% with prophylactic coagulation, with an overall rate of 6.6% [28]. That study also noted that CSDB is generally around 7% and may approach approximately 12% in proximal lesions.

Lesion size emerged as the only independent predictor of DB in multivariable analysis (OR 1.09 per mm, *p* = 0.001), while right-sided location showed a non-significant trend toward increased risk. These findings are consistent with prior large studies identifying lesion size and proximal colon location as key risk factors for DB and CSDB [30,31]. Importantly, the choice of submucosal injectate (TXA versus epinephrine) was not independently associated with DB, supporting the conclusion that TXA was not associated with an increased bleeding risk in this study.

The absence of a detectable reduction in CSDB with TXA in our cohort—despite its favorable tolerability profile—may be related to the relatively modest TXA dose used. Our protocol delivered an estimated 200–300 mg TXA submucosally in most cases, which is substantially lower than topical doses used in other surgical disciplines where consistent hemostatic benefits have been demonstrated.

The TXA dose used in this study was intentionally conservative. The injectate concentration was approximately 10 mg/mL, which is within ranges considered safe for soft tissues based on experimental and clinical data [32]. In other clinical contexts, substantially higher topical or systemic doses have been used. In orthopedic surgery, topical doses of 1–3 g TXA are commonly applied and have been shown to reduce blood loss without increasing thromboembolic risk [33,34]. Systemic dosing regimens in trauma and obstetric settings typically involve gram-level doses and have demonstrated survival benefits without excess thrombotic complications [35]. By comparison, the lower local doses used in our study may have limited the ability to detect differences in DB outcomes. This observation indicates that further studies evaluating alternative dosing strategies may be informative for assessing the hemostatic potential of TXA in the EMR setting.

TXA was well-tolerated in our cohort. No thromboembolic events, seizures, anaphylaxis, or other drug-related adverse events were observed, despite inclusion of patients with cardiovascular comorbidities and frequent antiplatelet exposure. This finding is consistent with extensive safety data from other medical specialties and supports the safety of TXA in the endoscopic setting [33,35], although the limited number of patients in this pilot study must be acknowledged.

Several baseline imbalances between groups should be acknowledged. Patients in the TXA arm were older, had higher BMI, more frequent ischemic heart disease, higher rates of continued aspirin use, and a greater prevalence of severe fibrosis. These factors could theoretically increase bleeding risk; however, none were independently associated with DB in multivariable analysis, and bleeding rates were numerically lower in the TXA group, suggesting that these baseline differences are unlikely to explain the observed findings.

This study has several limitations. It was a single-center pilot trial with a relatively small sample size and was conducted in a tertiary referral center with experienced endoscopists, which may limit generalizability. Only a single TXA dosing strategy was evaluated, precluding conclusions regarding optimal dosing or delivery methods. Nor was TXA compared against a saline or placebo control arm. In addition, the trial was not powered to detect uncommon adverse events such as thromboembolism or seizures, and the absence of such events should be interpreted with caution. Continuation of antiplatelet therapy was imbalanced between groups, being more frequent in the TXA arm, and this imbalance is acknowledged as a potential confounder. The small number of DB events also limited the multivariable analysis and increased the risk of model overfitting, so adjusted estimates should be regarded as exploratory. Finally, baseline differences were present between the groups, although adjusted analyses did not demonstrate an effect on primary outcomes.

## 5. Conclusions

In this randomized controlled pilot study, submucosal TXA was associated with less post-procedural pain and opioid use compared with epinephrine. Bleeding outcomes were comparable, although this pilot study was not powered to detect rare adverse events. Although TXA did not reduce DB at the dose studied, its favorable tolerability profile suggests that TXA may represent a potential alternative to epinephrine in submucosal injection during large colorectal EMR. Larger multicenter studies are warranted to confirm these findings, evaluate dose–response relationships, and determine the potential role of higher topical doses of TXA in improving EMR outcomes.

## Figures and Tables

**Table 1:** Baseline demographic characteristics of the study population.

	All (n = 121)	TXA, 60 (49.6%)	EPI, 61 (50.5%)	*p*-Value
Age (years), median [IQR]	69 [62, 75]	71 [65, 76]	67 [60, 72]	0.014
Male, n (%)	65 (53.7)	34 (52.3)	31 (50.8)	0.519
BMI, mean ± SD	27.2 ± 4.7	28 ± 4.7	26.4 ± 4.6	0.045
Smoker, n (%)	33 (27.3)	13 (21.7)	20 (32.8)	0.170
IHD, n (%)	16 (13.2)	12 (20)	4 (6.6)	0.034
DM, n (%)	33 (27.3)	21 (35.6)	12 (19.7)	0.066
COPD, n (%)	9 (7.4)	6 (10)	3 (4.9)	0.323
HTN, n (%)	64 (52.9)	34 (56.7)	30 (49.2)	0.409
Family history CRC, (%)	11 (9.1)	4 (6.7)	7 (11.5)	0.358

TXA = Tranexamic acid; EPI = Epinephrine; SD = standard deviation; BMI = Body mass index [kg/m^2^]; IHD = ischemic heart disease; DM = Diabetes mellitus; COPD = Chronic obstructive pulmonary disease; HTN = Hypertension; CRC = Colorectal Cancer.

**Table 2:** Antiplatelet treatment.

Antiplatelet Treatment	All	TXA	EPI	*p*-Value
Stop or without antiplatelet therapy during the procedure, n (%)	94 (77.7)	41 (68.3)	53 (86.9)	0.014
Continuous antiplatelet therapy, n (%)	27 (22.3)	19 (31.7)	8 (13.1)	
Anticoagulants, n (%)	13 (10.7)	7 (11.7)	6 (9.8)	0.754

TXA = Tranexamic acid; EPI = Epinephrine.

**Table 3:** Lesion characteristics according to study group.

	All	TXA	EPI	*p*-Value
Size (mm), median [IQR]	30 [25, 40]	30 [25, 40]	30 [20, 40]	0.908
**Location, n (%)**				0.492
Right colon	77 (63.6)	40 (66.7)	37 (60.7)	
Left colon + rectum	44 (36.4)	20 (39.3)	24 (39.3)	
**Paris classification, n (%)**				0.309
IS	23 (19)	13 (21.7)	10 (16.4)	
IIA	64 (52.9)	34 (56.7)	30 (49.2)	
IIB	2 (1.7)	1 (1.7)	1 (1.6)	
IIA + IS	31 (25.6)	11 (18.3)	20 (32.8)	
IIA + IIC	1 (0.8)	1 (1.7)	0 (0)	
**Morphology, n (%)**				0.741
Granular	83 (68.6)	42 (70)	41 (67.2)	
Non-Granular	38 (31.4)	18 (30)	20 (28.8)	
**JNET, n (%)**				0.089
I	12 (9.9)	3 (5)	9 (14.8)	
IIa	101 (83.5)	54 (90)	47 (77)	
IIb	7 (5.8)	2 (3.3)	5 (8.2)	
III	1 (0.8)	1 (1.7)	0 (0)	
**Pathology, n (%)**				0.921
SSP	16 (13.2)	6 (10)	10 (16.4)	
TA LGD	40 (33.1)	20 (33.3)	20 (32.8)	
TA HGD	19 (15.7)	9 (15)	10 (16.4)	
TVA LGD	16 (13.2)	9 (15.0)	7 (11.5)	
TVA HGD	24 (19.8)	13 (21.7)	11 (18)	
Malignant polyp	6 (5)	3 (4.9)	3 (5.0)	

IQR = Interquartile range; TXA = Tranexamic acid; EPI = Epinephrine; JNET = Japan NBI Expert Team classification; SSP = Sessile serrated polyp; TA = Tubular adenoma; LGD = low-grade dysplasia; HGD = high-grade dysplasia; TVA = Tubulo-villous adenoma.

**Table 4:** Procedural Features of Endoscopic Mucosal Resection Stratified by Submucosal Injectate.

	All (n = 121)	TXA, 60	EPI, 61	*p*-Value
Previous attempted, n (%)	14 (11.6)	10 (16.7)	4 (6.6)	0.082
Previous biopsy, n (%)	34 (28.1)	18 (30)	16 (26.2)	0.645
Presence of tattoo, n (%)	21 (17.4)	6 (10)	15 (24.6)	0.053
Resection type en-bloc, n (%)	21 (17.4)	10 (16.7)	11 (18.0)	0.843
Number of injections (mL), median [IQR]	20 [10, 30]	20 [10, 30]	20 [10, 30]	0.574
Fibrosis, n (%)	26 (21.4)	16 (26.7)	10 (16.4)	0.113
Severe fibrosis, n (%)	13 (10.7)	10 (16.7)	3 (4.9)	0.044
CAST, n (%)	10 (8.3)	7 (11.7)	3 (4.9)	0.205

IQR = Interquartile range; TXA = Tranexamic acid; EPI = Epinephrine; CAST = Cold-forceps Avulsion with Snare-Tip soft coagulation.

**Table 5:** Bleeding and Patient Outcomes.

	All	TXA	EPI	*p*-Value
**Bleeding during procedure**, n (%)				0.264
No	86 (71.1)			
Mild	23 (19)	11 (18.3)	12 (19.7)	
Moderate	11 (9.1)	8 (13.3)	3 (4.9)	
Severe	1 (0.8)	1 (1.7)	0 (0)	
**Control bleeding**, n (%)				
Spontaneous	7 (5.8)	5 (8.3)	2 (3.3)	0.475
STSC	25 (20.7)	13 (21.7)	12 (19.7)	
Perforation or deep injury	4 (3.3)	0 (0)	4 (6.6)	0.119
Pain after procedure	27 (22.3)	3 (5)	24 (39.3)	<0.001
Pain requiring opioids	11 (9.1)	0 (0)	11 (18)	<0.001
Delayed bleeding	12 (9.9)	5 (8.3)	7 (11.5)	0.762
Clinically significant delayed bleeding	8 (6.6)	4 (6.7)	4 (6.6)	>0.999
Clinically significant event	12 (9.9)	4 (6.7)	8 (13.1)	0.235
Post-polypectomy hospitalization	4 (3.3)	0 (0)	4 (6.6)	0.119
Follow up colonoscopy (6 months)	83 (68.6)	46 (76.7)	37 (60.7)	0.058
Recurrence	3 (3.6)	3 (6.5)	0 (0)	0.250

TXA = Tranexamic acid; EPI = Epinephrine; STSC = Snare Tip Soft Coagulation.

## Data Availability

The data supporting the findings of this study are not publicly available due to privacy and ethical restrictions. De-identified data may be made available from the corresponding author upon reasonable request, subject to institutional approval.

## Abbreviations

The following abbreviations are used in this manuscript:

ASGE	American Society for Gastrointestinal Endoscopy
BMI	Body mass index
CAST	Cold-forceps avulsion with snare-tip soft coagulation
CI	Confidence interval
COPD	Chronic obstructive pulmonary disease
CRC	Colorectal cancer
CSDB	Clinically significant delayed bleeding
CT	Computed tomography
DB	Delayed bleeding
DM	Diabetes mellitus
EMR	Endoscopic mucosal resection
EPI	Epinephrine
ESD	Endoscopic submucosal dissection
HGD	High-grade dysplasia
HTN	Hypertension
IHD	ischemic heart disease
IQR	Interquartile range
JNET	Japan NBI Expert Team classification
LGD	Low-grade dysplasia
NBI	Narrow-band imaging
NCT	National Clinical Trial number (ClinicalTrials.gov identifier)
NRS	Numerical rating scale
OR	Odds ratio
SD	Standard deviation
SSP	Sessile serrated polyp
STSC	Snare-tip soft coagulation
TA	Tubular adenoma
TVA	Tubulovillous adenoma
TXA	Tranexamic acid
